# Modeling and antitumor studies of a modified L–penetratin peptide targeting E2F in lung cancer and prostate cancer

**DOI:** 10.18632/oncotarget.26064

**Published:** 2018-09-07

**Authors:** Tazeem Shaik, Gulam M. Rather, Nitu Bansal, Tamara Minko, Olga Garbuzenko, Zoltan Szekely, Emine E. Abali, Debabrata Banerjee, John E. Kerrigan, Kathleen W. Scotto, Joseph R. Bertino

**Affiliations:** ^1^ Rutgers Cancer Institute of New Jersey, Rutgers, The State University of New Jersey, New Brunswick, NJ, USA; ^2^ Department of Pharmaceutics, Ernest Mario School of Pharmacy, Rutgers: The State University of New Jersey, Piscataway, NJ, USA; ^3^ Department of Biochemistry & Molecular Biology, Robert Wood Johnson Medical School, Rutgers, The State University of New Jersey, Piscataway, NJ, USA; ^4^ Department of Pharmacology, Robert Wood Johnson Medical School, Rutgers, The State University of New Jersey, Piscataway, NJ, USA; ^5^ Information Technology Division of Life Sciences and Chemistry, Rutgers School of Arts and Sciences, Rutgers, The State University of New Jersey, Piscataway, NJ, USA; ^6^ Department of Medicine, Robert Wood Johnson Medical School, Rutgers, The State University of New Jersey, Piscataway, NJ, USA

**Keywords:** E2F, D-Arg peptide, cytotoxicity, DU145, modeling

## Abstract

E2F1-3a overexpression due to amplification or to mutation or loss of the retinoblastoma gene, induces genes involved in DNA synthesis and leads to abnormal cellular proliferation, tumor growth, and invasion. Therefore, inhibiting the overexpression of one or more of these activating E2Fs is a recognized target in cancer therapeutics. In previous studies we identified by phage display, a novel 7-mer peptide (PEP) that bound tightly to an immobilized consensus E2F1 promoter sequence, and when conjugated to penetratin to increase its uptake into cells, was cytotoxic to several malignant cell lines and human prostate and small cell lung cancer xenografts. Based on molecular simulation studies that showed that the D-Arg penetratin peptide (D-Arg PEP) secondary structure is more stable than the L-Arg PEP, the L-Arg in the peptide was substituted with D-Arg. In vitro studies confirmed that it was more stable than the L- form and was more cytotoxic as compared to the L-Arg PEP when tested against the human castrate resistant cell line, DU145 and the human lung cancer H196 cell line. When encapsulated in PEGylated liposomes, the D-Arg-PEP potently inhibited growth of the DU145 xenograft in mice. Our findings validate D- Arg PEP, an inhibitor of E2F1and 3a transcription, as an improved second generation drug candidate for targeted molecular therapy of cancers with elevated levels of activated E2F(s).

## INTRODUCTION

Tumor and normal cell proliferation is mainly controlled by signals from the microenvironment which either stimulate or inhibit cell proliferation. Functionally, E2F1, E2F2, and E2F3a represent growth-promoting transcription factors and overexpression of E2F1 and/or 3 has been documented in various human cancers, including diffuse large cell lymphoma (DLCL), head and neck carcinoma, invasive ductal breast carcinoma, non-small cell lung carcinoma and prostate cancer [1–8]. Furthermore, high levels of E2F1 are associated with advanced disease and poor prognosis [8]. Deregulated E2F1 can induce angiogenesis, invasion, and metastasis resulting in more aggressive tumors [9].

The development of castrate resistant prostate cancer involves activation of an E2F1 mediated cell cycle network, implicating E2F1 as a key player in the process [7,10]. Loss of Rb during cancer progression correlated with increased levels of “free” E2F1 and androgen receptor (AR) levels in patients with castrate-resistant prostate cancer metastases [7]. Therefore, E2F1 plays a significant role as an oncogene and regulates multiple downstream targets relating to the cell cycle and proliferation [8,10–12].

Recently we reported that a novel peptide coupled to penetratin (PEP), was cytotoxic at low micro molar concentrations to tumors that overexpress activating E2Fs, including Burkitt lymphoma cells, pRB negative small cell lung cancer (SCLC) cells and DU145 prostate cancer cells. This unique E2F1 inhibitory peptide with the sequence His-His-His-Arg-Leu-Ser-His, found by phage display, was discovered by its ability to bind tightly to an immobilized consensus E2F1 promoter sequence [10]. Importantly, treatment of tumor xenografts of human small cell lung cancer H69 and human prostate cancer DU145 propagated in mice with a more stable PEGylated liposome encapsulated penetratin peptide (PL-PEP) caused tumor regression without significant toxicity [10,13].

In general, peptides are rapidly degraded by serum proteases. However, there are a vast array of modifications for protecting biologically active peptides from enzymatic degradation, such as alteration of the amide bond, N terminal acetylation, C terminal amidation or methylation, head to tail cyclization, incorporation of non-natural amino acids such as beta- or D- amino acids, as well as inclusion of structural constraints such as disulfide bonds [14–16]. As the E2F inhibiting L-enantiomeric penetratin peptide was found to be unstable in serum, based on modeling studies (*vide infra*) a new peptide form, D- Arg PEP was generated by substituting L-arginine with D-arginine in the L- PEP peptide sequence. In this study we assessed the stability, antitumor efficacy of the D- Arg penetratin peptide (D- Arg PEP) against H196 SCLC cells, DU145 prostate cancer cells, and the anti-tumor efficacy of PEGylated liposome encapsulated PEPs against DU145 prostate xenografts in nude mice.

## RESULTS

### Modeling of L/D-Arg PEP stability

Overall the simulations attempt to evaluate the stability of the peptides to an aqueous environment at 300 K and 1 atm. There are two levels of comparison when reviewing the plots that follow. One comparison is between the L-Arg PEP peptide and its D-Arg counterpart, D-Arg-PEP. The substitution of the L-Arg residue with D-Arg was made to better protect this peptide from trypsin cleavage. The other level of comparison is between the non-protected peptides (fully charged N and C-terminal groups) versus the N and C-terminal protected peptides described in computational methods.

**Figure 1:**
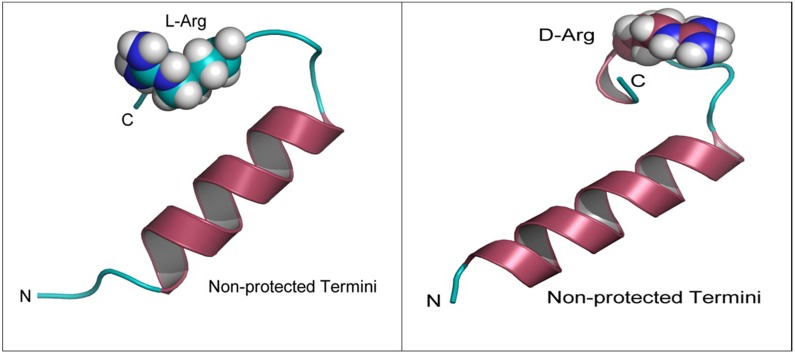
Modeling of D-Arg-PEP and L-Arg-PEP Peptide structures labeled by N and C termini after 20 ns of molecular dynamics simulation in a TIP3P water box at 1 atm. Note the loss in secondary structure (α-helix) when comparing the D-Arg to L-Arg.

**Figure 2:**
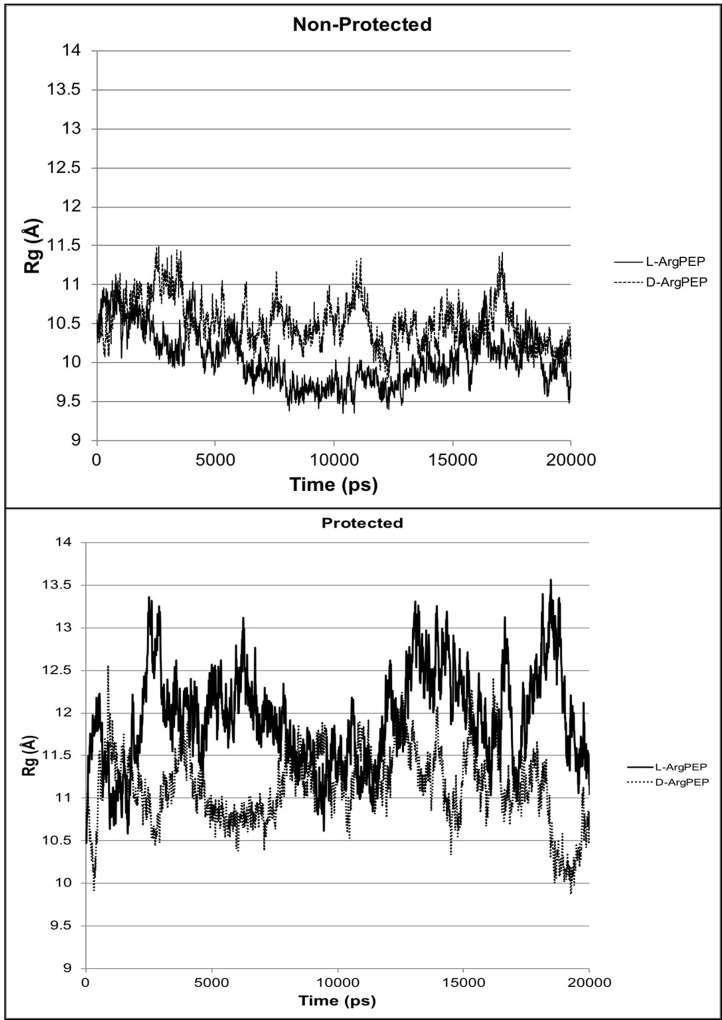
Radius of gyration Rg (Å) plots from molecular dynamics simulations for D-Arg-PEP and L-Arg-PEP comparing the protected termini with the non-protected termini for the peptides For the protected peptides (neutral termini), the N-terminus nitrogen is acetylated and the C-terminus is protected as the *N*-methyl amide. The non-protected peptides were modeled in their charged state (charged termini for pH 7.5).

In both the non-protected and protected forms, the peptide stability energies (Table 1) show that D-Arg PEP is the more stable of the two peptides. The structures (Figure 1) and the radius of gyration plots (Figure 2) indicate the non-protected peptides assume a more compacted form as the simulation progresses. In contrast, the protected peptides radius of gyration plots indicate these peptides do not progress to a more compact structure. The protected peptides show large variability in radius of gyration. A key requirement for binding to the DNA major groove is the stability of the α-helix structure of the penetratin peptide. The D-Arg substitution (Figure 1) clearly gives a more stable α-helix compared to L-Arg in the same position along the peptide chain. Protection of the N and C terminal ends of the peptide destabilizes the α-helix in both the L-Arg and D-Arg peptides and therefore the protected peptides are predicted to be less effective binders.

**Table 1:** Peptide stability energies of the peptides in kcal/mol

Peptide	*∆H*stability (kcal/mol)	∆∆*H* rel (kcal/mol)
**L-ArgPEP**	−731.4 ± 14.9	+11.2
**D-ArgPEP**	−742.6 ± 16.6	0.0
**(P)L-ArgPEP**	−598.0 ± 16.9	+14.5
**(P)D-ArgPEP**	−612.5 ± 16.1	0.0

Note: The (P) designates “Protected”. ∆∆H _rel_ = E(DARG) - E(Pep). Overall, the non-protected peptides are predicted to be more stable in water than the protected peptides.

### D- Arg PEP has more potent anti-proliferative activity compared to the L- Arg PEP

**Figure 3:**
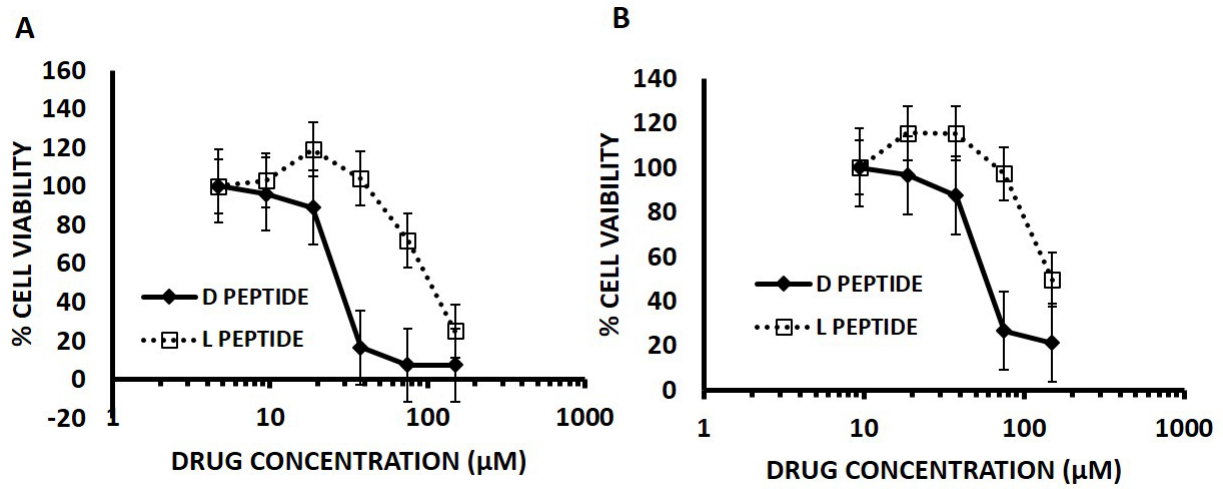
D- Arg Peptide is more effective than the L- Arg Peptide in (A) DU145 cells and (B) H196 SCLC cells at 24 hours of treatment In this assay, 5000 cells per well were plated for the 24-hour time point in a 96 well plate on day zero in RPMI media containing 10% FBS. After 24 hours either the D- Arg peptide or the L- Arg Peptide was added in serial dilutions across the plate. Cell viability was assessed at 24 hours of treatment by measuring the absorption at 490nm using the MTS tetrazolium Promega CellTitre 96^®^ Aqueous One Solution Cell proliferation assay according to the manufacturer’s instructions. Each time point was done in triplicate and values are represented by mean with standard mean deviation.

We compared the IC_50_ of D- Arg PEP with L- Arg PEP in DU145 prostate cancer and H196 small cell lung cancer cell lines (SCLC). Both cell lines lack pRb and have mutant p53, and have increased levels of E2F-1. The D-Arg PEP decreased cell viability in both cell lines with greater potency than the L- Arg PEP (Figure 3). Our previous studies showed that the L-Arg PEP caused apoptosis as the mechanism of cell growth inhibition [10].

### D- Arg PEP is resistant to inactivation by serum

**Figure 4:**
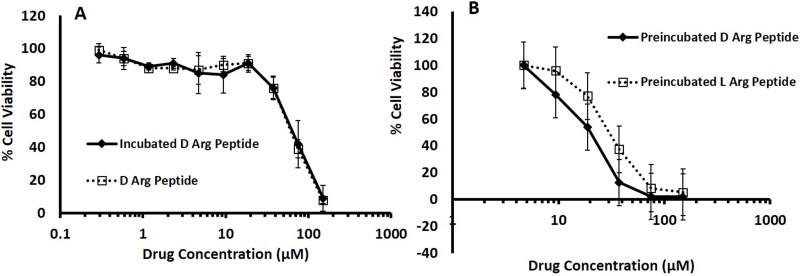
**A.** D- Arg Peptide incubated in 10% FBS RPMI media at 37°C for 24 hours is resistant to proteolysis; it is as effective as the non-incubated D- Arg Peptide in DU145 cells. D- Arg Peptide was incubated in RPMI media containing 10% FBS at 37°C for 24 hours. DU145 cells were seeded in a 96 well plate at 5000 cells in each well. After 24 hours either the incubated D- Arg Peptide or the non-incubated D- Arg Peptide was added to the cells. **B.** D- Arg and L-Arg Peptide incubated in 10% FBS RPMI media at 37°C for 24 hours. DU145 cells were seeded in a 96 well plate at 5000 cells in each well. After 24 hours either the incubated D- Arg Peptide or the L- Arg Peptide was added to the cells. The cell viability in both experiments were assessed after another 24 hours by measuring the absorption at 490nm using the MTS tetrazolium Promega CellTitre 96^®^ Aqueous One Solution Cell proliferation assay according to the manufacturer’s instructions. Each time point was done in triplicate and values are represented by mean with standard mean deviation.

To confirm the increase in stability of the D- Arg PEP compared to the L-Arg PEP, we incubated the D- Arg PEP in RPMI 1640 media containing 10% FBS for 24 hours and compared the toxicity of the incubated with fresh non-incubated D- Arg PEP against the DU145 cell line. The pre-incubated D- Arg peptide was as potent as the non-incubated fresh D- Arg PEP, indicating that the D- Arg-PEP was stable in FBS and media (Figure 4A). The incubated D- Arg peptide was also compared with the incubated L- Arg peptide after 24 hours of treatment. The D- Arg PEP was more resistant to proteolysis after pre-incubation in serum, unlike the L- Arg PEP as shown in Figure 4B.

### PEGylated Liposomal Encapsulation of the D- Arg Peptide inhibited growth of DU145 xenografts *in vivo*

**Figure 5:**
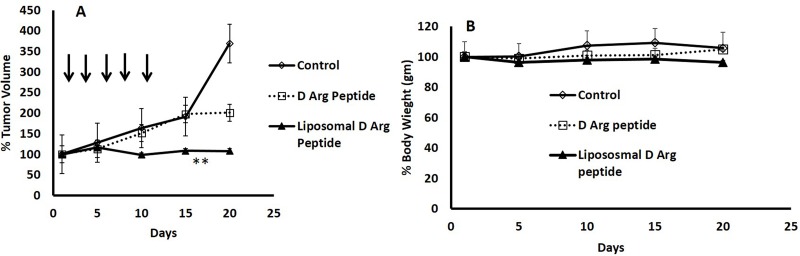
PEGylated Liposomal Encapsulation of the D- Arg Peptide inhibited growth of DU145 xenografts in mice 2 million DU145 cells were injected subcutaneously in the abdominal flanks of nude mice. Once the tumor was palpable, mice were randomized into 3 groups (*n* = 4). Mice were injected then with D- Arg peptide (60mg/Kg) or with Liposomal D- peptide (100mg/Kg) every other day for 10 days. Control mice received saline. The Liposomal D-Arg peptide caused marked inhibited growth of the DU145 in mice compared to D-Arg peptide or control **A.**, without causing weight loss or signs of toxicity **B.** Tumor size was measured every other day; tumor volume was measured using the formula (length x width^2^)/2. Data was plotted and SEM was calculated. **p-value equal to 0.05.

As the D- Arg PEP was stable in FBS/RPMI media, we compared the anti-tumor activity of the D- Arg PEP with the D- Arg PEP encapsulated in PEGylated liposomes against DU145 xenografts. As shown in Figure 5A, after two weeks of every other day treatment, the liposome encapsulated D- Arg PEP caused marked growth inhibition. The non-encapsulated L-Arg PEP administered at the same dose and schedule was less effective. There is no weight loss or other evidence of toxicity (Figure 5B).

## DISCUSSION

Many studies have documented E2F1 overexpression in various human cancers causing tumor progression, invasion and metastases [8–12]. This drew our attention to target based therapy against E2F1, given its role in G_1_-S transition via activating downstream cell regulating genes (e.g., thymidylate synthase (TS), dihydrofolate reductase (DHFR), cyclin A, and cyclin E) [17] and cellular mechanisms which are crucial for proliferation, malignant transformation, invasion; and resisting apoptosis through upregulation of proteins such as bcl-2 [18]. Thus inhibiting E2F1 activation/overexpression would have a therapeutic potential. We tested this hypothesis in previous studies with a novel 7-mer peptide coupled to penetratin (PEP) which is an L- enantiomeric peptide [10,13].

Current research in molecular cancer therapeutics is aimed at the discovery and development of new peptide drugs with superior activity. Cellular uptake, bioavailability, *in vivo* activity, cytotoxicity, proteolytic degradation as well as clearance, and adverse effects are some of the important aspects taken into account while developing a good peptide drug candidate. Albeit rapidly degraded by proteases, peptides are attractive molecules due to their low toxicity and unparalleled specificity to a range of targets. Human serum proteases recognize some peptides composed of L-amino acids, and decrease their biological activity by causing proteolysis. Thus developing a peptide with increased serum stability is fundamental and demanding. Chemical modifications of synthetic peptides are a very common means of controlling their functions. Therefore, to enhance the stability and antitumor activity of the PEP, we modified the penetratin peptide by substitution of a D- Arg PEP from the L-amino acid, as certain serum peptidases recognize L-Arg coupled to adjacent amino acids synthesized [19].

D-peptides have several advantages over L-enantiomeric peptides. The isomerization of an L- to D-amino acid is a remarkable post-translational modification of peptides in RNA-based protein synthesis essential for biological function and has been documented in amphibians, invertebrates, and mammals [19]. Studies have shown that all D-amino acid analogs have identical chemical and physical properties but possess different biological activities relative to natural all L-peptides; this property has been utilized in designing antimicrobial peptides that can resist proteolytic degradation [14,20]. In many cases, the D-amino acid containing peptides exhibit dramatically higher affinity and selectivity for receptor binding than their all-L counterparts and thus superior in biological function [21]. Peptides that are at least partially substituted by corresponding D-amino acids are more stable and strongly resistant to proteolytic degradation, greatly increasing serum and saliva half-life of the peptide [19,20]. Additionally, D-peptides owing to their ability to resist degradation by digestive proteolytic enzymes, can be absorbed systemically after oral administration (with the efficiency determined by the sequence), in contrast to L-peptides, which have to be injected to avoid digestion [20]. D-peptides have long shelf-lives and because they are chemically synthesized, they can easily be modified [20]. Therefore, substitution of L- by D-amino acids is a well-established strategy to increase the stability of a peptide [21–23].

Because of the advantages described above, we examined the effect of substituting L-Arginine with D-Arginine on the stability and activity of the E2F-penetratin peptide. Replacement of D versus L amino acids increased the peptide’s anti-proliferative activity against DU145 prostate cancer cells and H196 small cell lung cancer cells.

While our studies showed an advantage of liposomal encapsulation of the D-Arg PEP over the non-encapsulated Arg-PEP, more frequent dosing of the latter preparation may increase its antitumor effects. The lack of toxicity of the liposomal PEP noted in previous studies and the current study is worthy of note. A recent study comparing the effects of knockdown of E2F1 in chronic myelocytic leukemia stem cells and normal marrow stem cells showed that unlike CML stem cells, normal marrow stem cells were unaffected by the knockdown of E2F1 [24].

Inhibition of transcription of E2F1, results in downstream regulation of proteins that are targets for chemotherapeutic drugs including pemetrexed which targets TS to decrease its expression; and hydroxyurea which targets ribonucleotide reductase (RR) [10,13,25]. Overexpression of bcl-2, a known antiapoptotic oncogene, seems to be critical in transforming prostate cancer cells from an androgen-dependent to an androgen-independent state [26]. E2F1 resists apoptosis through upregulation of bcl-2 [18] and E2F1 inhibition by our novel anticancer peptide is hypothesized to promote apoptosis in part through the downregulation of bcl-2. Future studies will test the combination of the D-Arg PEP with inhibitors of DHFR, TS and Bcl-2.

## MATERIALS AND METHODS

### Animals

Experiments were conducted in accordance with the Rutgers Cancer Institute of New Jersey Animal Care and Use Committee guidelines (protocol number I13-003). Nude mice were obtained from the Jackson Laboratory (Bar Harbor, ME).

### Cell lines and chemical compounds

Prostate cancer (DU145) and Lung cancer cell lines (H196) were obtained from the ATCC (Manassas, Virginia) and maintained free of Mycoplasma. H196 cells were cultured in the modified RPMI 1640 medium (ATCC Catalog No. 30-2001) and the DU145 cells were cultured in the RPMI 1640 medium (Life technologies, Grand Island, NY) with 10% FBS (Life Technologies, Grand Island, NY), 1% penicillin/streptomycin (Life Technologies, Grand Island, NY) in an atmosphere of 5% CO2. D- Arg PEP was synthesized by Bio Basic Inc., Ontario, Canada and validated by the Rutgers Chemistry Core as previously described [10].

### Computational methods

All calculations were performed on an HP Z820 workstation equipped with dual 8-core Intel Xeon processors and an NVidia Tesla GPU card. The Amber 12 suite of biomolecular simulation programs was used for all molecular mechanics and dynamics calculations [27]. Molecular dynamics production runs were performed using the Amber PMEMD CUDA gpu optimized program. The Amber ff12SB force field was used for all calculations. The penetratin-linked peptides (e.g. peptide BRT1 is RQIKIWFQNRRMKWKKHHHRLSH) were built from 9ANT.pdb (template) [28] using the Modeller program [29, 30]. The N-terminal (ACE or acetyl) and C-terminal (NME or *N*-methyl amide) protected models were built from the homology models using the Sybyl (Tripos) molecular modeling software package. Each model was energy minimized in vacuo followed by encapsulation in an octahedral periodic box of TIP3P water [31] using a 12 Å spacing. Overall positive formal charge in each system was neutralized by replacement of water molecules by the appropriate number of chloride ions. The default 8 Å cutoff was used for non-bonded interactions. The PME method was used to account for long-range electrostatic interactions [32,33]. A Langevin thermostat was used with a collision frequency γ = 5 s^-1^ and random number seed [34] to control temperature in the system to 300 K. A Berendsen barostat was used to keep pressure at 1 atm [35]. The models were energy minimized keeping the peptide atoms restrained (500 steps conjugate gradient) followed by energy minimization without restraints on the system. A 50 ps simulation holding the peptide restrained with temperature at 300 K was performed using constant temperature and constant volume (NVT). This step was followed by another 50 ps simulation keeping peptide atom positions restrained using constant temperature and constant pressure (1 atm). The system was run at 300 K and 1 atm (NPT) for a 20 ns production run. All trajectory analysis was performed using either the Amber ptraj or cpptraj programs [36]. The stability energies were estimated from 100 evenly spaced snapshots (stripped of water and ions) from the last 1 ns of production run using the generalized Born OBC II implicit solvent model with a salt concentration of 0.1 M [37]. Illustrations were prepared using the Pymol (Delano, W; Schrodinger LLC) molecular graphics software tool.

L-Arg PEP Penetratin-HHHRLSH
D-Arg PEP Penetratin-HHH(D)RLSH

### Cell viability assays

5000 cells per well were plated in a 96 well plate in 180 μl of RPMI 1640 media containing 10% FBS. After 24 hours, 20 μl of either L- or D- Arg Peptide was added and incubated for 24 hours. 20 μl of the MTS tetrazolium Promega CellTitre 96^®^ Aqueous One Solution Cell Proliferation Assay (Promega, Madison, WI) was added to each well and incubated for two to three hours. Absorbance was measured at 490 nm as per the manufacturer’s protocol to determine the cell viability.

### Stability testing of the peptide

The peptide was mixed with the RPMI 1640 medium containing 10% FBS and incubated at 37oC for 24 hours. Cells were plated in a 96 well plate in 180 μl of the media containing 10% FBS. After 24 hours, 20 μl of either the incubated D- Arg or the regular (fresh) D- Arg Peptide was added to the cell plate and tested after another 24 hours. Then 20 μl of the MTS tetrazolium Promega CellTitre 96® Aqueous One Solution Cell Proliferation Assay was added to each well and incubated for two to three hours. Absorbance was measured at 490 nm as per the manufacturer’s protocol to determine the cell viability.

### Xenograft studies

Two million DU145 cells were injected subcutaneously in the abdominal flanks of nude mice. Once the tumor was palpable, mice were randomized into 3 groups (*n* = 4). Mice were injected then with D- Arg peptide (60mg/Kg) or with Liposomal D- Arg peptide (100mg/Kg) every other day for 10 days. Control mice received saline. Tumor size was measured every other day and tumor volume was measured using the formula (length x width^2^)/2. Data was plotted and SEM was calculated.

### Statistical analysis

All *in vitro* experiments were performed three times, and each experiment was done in triplicate. Statistical analysis was performed using GraphPad Prism software. In all cases, ANOVA followed by two-tailed, unpaired Student t tests were performed to analyze statistical differences between groups. *P* values of <0.05 were considered statistically significant.
